# Erythrocyte G Protein as a Novel Target for Malarial Chemotherapy

**DOI:** 10.1371/journal.pmed.0030528

**Published:** 2006-12-26

**Authors:** Sean C Murphy, Travis Harrison, Heidi E Hamm, Jon W Lomasney, Narla Mohandas, Kasturi Haldar

**Affiliations:** 1 Department of Pathology, Feinberg School of Medicine, Northwestern University, Chicago, Illinois, United States of America; 2 Department of Microbiology-Immunology, Feinberg School of Medicine, Northwestern University, Chicago, Illinois, United States of America; 3 Department of Pharmacology, Vanderbilt University Medical Center, Nashville, Tennessee, United States of America; 4 Department of Molecular Pharmacology and Biological Chemistry, Feinberg School of Medicine, Northwestern University, Chicago, Illinois, United States of America; 5 New York Blood Center, New York, New York, United States of America; Imperial College, United Kingdom

## Abstract

**Background:**

Malaria remains a serious health problem because resistance develops to all currently used drugs when their parasite targets mutate. Novel antimalarial drug targets are urgently needed to reduce global morbidity and mortality. Our prior results suggested that inhibiting erythrocyte G_s_ signaling blocked invasion by the human malaria parasite *Plasmodium falciparum.*

**Methods and Findings:**

We investigated the erythrocyte guanine nucleotide regulatory protein G_s_ as a novel antimalarial target. Erythrocyte “ghosts” loaded with a G_s_ peptide designed to block G_s_ interaction with its receptors, were blocked in β-adrenergic agonist-induced signaling. This finding directly demonstrates that erythrocyte G_s_ is functional and that propranolol, an antagonist of G protein–coupled β-adrenergic receptors, dampens G_s_ activity in erythrocytes. We subsequently used the ghost system to directly link inhibition of host G_s_ to parasite entry. In addition, we discovered that ghosts loaded with the peptide were inhibited in intracellular parasite maturation. Propranolol also inhibited blood-stage parasite growth, as did other β_2_-antagonists. β-blocker growth inhibition appeared to be due to delay in the terminal schizont stage. When used in combination with existing antimalarials in cell culture, propranolol reduced the 50% and 90% inhibitory concentrations for existing drugs against P. falciparum by 5- to 10-fold and was also effective in reducing drug dose in animal models of infection.

**Conclusions:**

Together these data establish that, in addition to invasion, erythrocyte G protein signaling is needed for intracellular parasite proliferation and thus may present a novel antimalarial target. The results provide proof of the concept that erythrocyte G_s_ antagonism offers a novel strategy to fight infection and that it has potential to be used to develop combination therapies with existing antimalarials.

## Abstract 2

Erythrocyte G protein signaling is needed for intracellular malarial parasite proliferation and thus may present a novel antimalarial target.

## Editors' Summary

**Background.:**

New drugs for treatment of malaria are urgently needed, because the malaria parasite has evolved resistance against virtually all types of commonly used drugs. When a person is bitten by a malaria-infected mosquito, the parasite first infects the person's liver cells before going on to infect red blood cells, where the parasites multiply and develop into a parasite stage called a schizont. The red blood cells then burst and release more schizonts into the bloodstream; it is this “blood stage” of infection in humans that causes the symptoms of disease. Therefore efforts to develop new drugs against malaria often focus on this “blood stage” of infection. One strategy for developing new drugs is termed the “host-targeted” approach. This means that rather than trying to block processes occurring within the parasite itself, a drug can be developed which blocks processes within the person's red blood cells, and which would otherwise be needed for the parasite to complete its life cycle. It will be difficult for malaria parasites to evolve resistance to such a drug, because changes in a person's red blood cells occur much more slowly than in the parasites themselves.

**Why Was This Study Done?:**

This research group has been studying a set of molecular processes within human red blood cells which seemed to be required for entry of malaria parasites into the cells. They wanted to get a better understanding of those processes and, specifically, to find out whether it would be possible to use particular molecules to block those processes, and by doing so to prevent malaria parasites from entering and multiplying within red blood cells. In particular, when the malaria parasites invade the red blood cell, they form membranes around the red blood cell, containing lipids and proteins “hijacked” from the red blood cell membrane. These researchers already knew that two particular proteins were hijacked in this way; the β_2_-adrenergic receptor (β_2_-AR) and heterotrimeric G protein (G_s_). These two proteins act together to pass messages across the surface of the membrane to inside the cell. Small molecules could be used to block signaling through β_2_-AR and G_s_, and therefore potentially to provide a new way of preventing malaria parasites from entering red blood cells and multiplying within them.

**What Did the Researchers Do and Find?:**

Firstly, the researchers made red blood cell “ghosts” in which to study these molecular processes. This meant that they took fresh red blood cells from healthy human volunteers, burst them to remove half the contents and loaded them with markers and other cargoes before resealing the membranes of the cell. These resealed markers and cargoes allowed them to see what was happening inside the cells. Malaria parasites were able to invade these ghosts normally and multiply within them. When the researchers introduced a specific peptide (a molecule consisting of a short series of amino acids), they found that it blocked G_s_ signaling within the ghosts. This peptide also prevented malaria parasites from developing inside the ghosts. Therefore, they concluded that G_s_ signaling inside the red blood cell was important for the parasite life cycle. The researchers then examined a drug called propranolol which is already known to act on G_s_ signaling and which is commonly prescribed for high blood pressure. This drug also blocked development of malaria parasites inside the ghosts when used at a particular concentration. Finally, the researchers studied the effect of giving propranolol, along with other antimalarial drugs, to human malaria parasites in a culture dish and to mice injected with a malaria parasite that infects rodents. In these experiments, adding propranolol reduced the amount of other “parasite-targeted” drugs that were needed to effectively treat malarial infection in tissue culture and in mice.

**What Do These Findings Mean?:**

Showing that the G_s_ signaling pathway is important for the malaria parasite's life cycle opens up new possibilities for drug development. Specifically, propranolol (which is already approved for treatment of high blood pressure and other conditions) might itself provide a new candidate therapy, either alone or in combination with existing drugs. These combinations would first, however, need to be tested in human clinical trials, perhaps by seeing whether they have antimalarial activity in people who have not responded to existing antimalarial drugs. Since it acts to lower blood pressure, which can already be low in some people with malaria, there are some concerns that propranolol might not be a suitable drug candidate for use, especially with existing antimalarial drugs that also reduce blood pressure. However, other molecules which block G_s_ signaling could be tested for activity against malaria should propranolol prove not to be an ideal drug candidate.

**Additional Information.:**

Please access these Web sites via the online version of this summary at http://dx.doi.org/10.1371/journal.pmed.0030528.

- The World Health Organization publishes a minisite containing links to information about all aspects of malaria worldwide, including treatment, prevention, and current programs for malaria control
- Medicines for Malaria Venture is a collaboration between public and private organizations (including the pharmaceutical industry) that aims to fund and manage the development of new drugs for treatment and prevention of malaria
- Wikipedia entries for drug discovery and drug development (Wikipedia is an internet encyclopedia that anyone can edit)

## Introduction

Plasmodium falciparum is a protozoan parasite that causes the most lethal form of malaria, a major human disease urgently in need of new targets for future antimalarial drugs. Targeting the blood stages of infection is particularly important since these stages are responsible for all of the symptoms and pathologies associated with the disease. Increased resistance to antimalarial drugs is inevitably linked to mutation of parasite targets and reflects the general problem of continual emergence of resistance to drugs developed against microbial targets. Indeed, the discovery of drugs and targets that minimize the likelihood of drug-resistant microorganisms was recognized as a Grand Challenge in Global Health [1]. One strategy is to target critical host determinants needed for infection.

During P. falciparum blood-stage infection, the extracellular merozoite adheres to erythrocytes, and invades and develops intracellularly, surrounded by a parasitophorous vacuolar membrane. Merozoites that do not invade die, and thus erythrocytic infection is critical for parasite proliferation. In contrast to most cells, mature erythrocytes do not possess active endocytic machinery. Thus, malarial invasion was long considered to be dependent mainly on parasite factors released from invasive organelles of merozoites (i.e., rhoptries, dense granules, micronemes) [2]. However, a recent study showed that components enriched in erythrocyte detergent-resistant membrane lipid rafts are also involved in invasion [3]. At least 25 erythrocyte proteins reside in lipid rafts, and 12 of these are recruited to the parasitophorous vacuolar membrane, including two well-studied signaling proteins: the β_2_-adrenergic receptor (β_2_-AR) and its associated heterotrimeric G protein, G_s_ ([4–7]; SCM and KH, personal communication).

Accordingly, Harrison and coworkers found that β-adrenergic receptor (β-AR) and adenosine-receptor agonists (isoproterenol and 5′-*N*-ethylcarboxamidoadenosine, respectively), stimulated malarial entry into erythrocytes, while the respective antagonists (propranolol and 8-(*p*-sulfophenyl)theophilline) reversed agonist-induced stimulation of entry [7]. However, *Plasmodium* is a eukaryote and shares many cellular mechanisms with its host. In addition, erythrocytes are terminally differentiated cells, their signaling mechanisms are not well understood, and direct evidence that erythrocyte G proteins are active is not available. Thus, the finding that a peptide designed to inhibit host G_s_ function by blocking its interaction with host G-protein–coupled receptors (GPCRs) reduced invasion was important to the idea that erythrocyte G_s_ signaling regulated malarial invasion, even though P. falciparum lacks conserved heterotrimeric G proteins [7]. However, the peptide does not enter uninfected erythrocytes, and hence there is no evidence that it blocks endogenous erythrocyte G_s_ signaling. Furthermore, invasion alone is not considered a strong target for antimalarials because it is rapid (occurring in minutes). Agents that block invasion have a narrow window in which to act on the parasite and hence may reduce parasitemia, but may fail to quickly eradicate the parasite. Whether host G_s_ is needed for intracellular parasite growth as well as invasion, and may thus present a target for antimalarials, was not known.

In this paper we describe the development of erythrocyte “ghosts” that are active in β-adrenergic signaling and which closely mimic normal erythrocytes in supporting P. falciparum growth in culture. We used this system to investigate cytoplasmic erythrocyte molecules important in erythrocyte function, malarial entry, and intracellular growth and to determine whether such host molecules may serve as useful targets for antimalarial drug development.

## Methods

### Materials

G_s_, G_scr_, G_i_, and G_q_ peptides were described previously [7], although G_s_ and G_scr_ peptides were obtained from the Protein Chemistry Core at Baylor College of Medicine (Houston, Texas, United States). All peptides were lyophilized and resuspended in culture medium before being added to cell cultures. Adenosine 5′-triphosphate (ATP), guanosine 5′-triphosphate (GTP), 70-kDa fluorescein isothiocyanate (FITC)–dextran, rhodamine-dextran, and drugs were from Sigma (http://www.sigmaaldrich.com). Lucifer yellow (LY), lucifer yellow-dextran (LY-D), and antibodies specific for glutathione *S*-transferase (GST, A5800) and green fluorescent protein (GFP, A6455) were from Invitrogen (http://www.invitrogen.com). Other chemicals were reagent-grade products from standard sources.

### Preparation of Erythrocyte Ghosts and Loading of Cargo

Non-outdated blood obtained from LifeSource (http://www.lifesource.org) or from consenting adult donors (Northwestern University Institutional Review Board approval number 1425–002) was washed and erythrocytes were resuspended to 50% hematocrit (Hct) in cold PBS-glucose containing 1 mM ATP and a variety of cargoes. FITC- or rhodamine-labeled 70-kDa dextran (final concentration 1.0–2.5 mg/ml) were used as surrogate loading markers when non-fluorescent cargoes were loaded. Samples were chilled on ice and dialyzed with stirring against ice-cold 5 mM K_2_HPO_4_ supplemented with 1 mM ATP (final pH 7.4) for 1 h at 4 °C in pre-wetted 3.5-kDa molecular weight cut-off (MWCO) Slide-a-lyzer dialysis cassettes (Pierce Biotechnology, http://www.piercenet.com). Lysis was performed in buffers ≤ 4 °C to prevent premature membrane resealing. Lysis of erythrocytes by dialysis and simultaneous equilibration with cargo avoided high-speed centrifugation of membranes. Use of a 3.5-kDa MWCO dialysis membrane minimized loss of small proteinaceous components of the erythrocyte cytoplasm.

**Figure 1:**
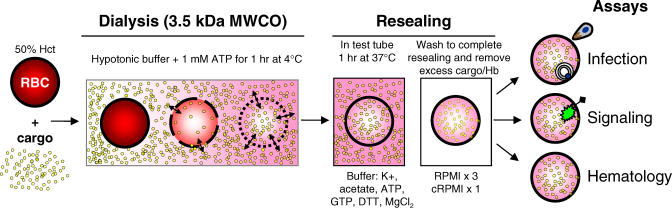
Schematic for the Production of Ghosts with G_s_ Signaling and Malarial Infection that Closely Mimic Normal Erythrocytes Erythrocytes were washed and resuspended to 50% Hct in PBS-glucose containing proteins of interest (“cargo,” yellow spheres) as described in Methods. The cell suspension was dialyzed against ATP-supplemented hypotonic potassium buffer, lysing cells, and allowing exogenous cargoes to enter and endogenous cytoplasmic proteins to exit lysed cells (depicted over time as increasing extracellular hemoglobin, change in cell color from red to pink, and loss of membrane integrity). Lysed cells were removed to cold test tubes and resealed at 37 °C for 1 h and washed three times in RPMI and once in RPMI supplemented with 10% human serum (cRPMI) to eliminate extracellular hemoglobin, cargo, and buffer (see Methods). Ghosts prepared in this manner were subsequently characterized in hematological, signaling, and malarial infection assays, as described in Figure 2.

Lysed erythrocytes were removed from dialysis membranes at 4 °C and were combined with one-fifth by volume of concentrated resealing buffer (475 mM KOAc, 25 mM Na_2_HPO_4_, 25 mM MgCl_2_, and 237.5 mM KCl [pH 7.0], stored at 25 °C, supplemented with 1.5 mM dithiothreitol [DTT], 1 mM ATP, and 5 mM GTP) and incubated for 1 h at 37 °C with rotating. The final ionic concentrations were 95 mM KOAc, 5 mM MgCl_2_, 5 mM Na_2_HPO_4_, 47.5 mM KCl, 0.3 mM DTT, 1 mM ATP, and 1 mM GTP. Potassium was favored in the resealing buffer because intracellular potassium concentration is high in normal, intact cells and a potassium-rich environment is known to be preferred by malaria parasites [8]. ATP (1 mM) and GTP (1 mM) were added to maintain critical nucleotide pools. Resealed ghosts were washed three times in serum-free RPMI-1640 (RPMI) at room temperature and resuspended to 10%–50% Hct in RPMI containing 10% human serum (complete RPMI-1640 [cRPMI]). Ghosts prepared in this manner could be centrifuged at low *g* forces (600 *g*), similar to normal erythrocytes. As indicated in Figure 1, they were used to evaluate hematological properties, signaling functions, and malarial infection. Reports indicate that resealed ghosts can be permeable to small molecules [9,10], so when small cargoes were used (<10 kDa), ghosts were continuously bathed with cargo during the assays. Single hematograms of normal and ghosted erythrocytes that provided mean corpuscular volume (MCV), mean corpuscular hemoglobin (MCH), and red cell distribution width (RDW) were obtained using an automated Coulter counter (Department of Pathology, Northwestern Memorial Hospital, Chicago, Illinois, United States).

**Figure 2:**
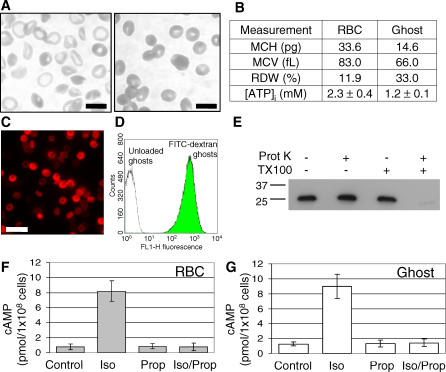
Hematological and Signaling Characteristics and Cargo Loading of Erythrocyte Ghosts (A) Ghosts (left) were biconcave but retained less pigment than intact erythrocytes (right); bar represents 10 μm. (B) Ghost MCH (normal range for erythrocytes 27.5–33.5 pg/cell), MCV (normal range for erythrocytes 80–100 fl), and RDW (normal range for erythrocytes 11.0%–15.0%) were determined using a Coulter counter (see Methods). Intracellular ATP levels were determined by luciferase-based ATP assays (see Methods); the 95% confidence interval (CI) for ATP levels is indicated; two experiments. (C) Ghosts loaded with high molecular weight rhodamine-labeled antibody were imaged without fixation by fluorescence microscopy; bar represents 25 μm. (D) Flow cytometry of unloaded (white fill) and FITC-dextran–loaded ghosts (green fill) 2 h post-resealing, showed homogenous loading of cargo. For each cell type, 100,000 gated events were captured. (E) Western blot of GST-loaded ghosts after incubation in culture for 24 h and subsequent treatment in the presence or absence of proteinase K (Prot K) and/or Triton X-100 (TX100); see Methods. GST was detected by GST-specific immunoblotting. (F) cAMP production in normal, intact erythrocytes treated with isopreterenol (iso) and/or propranolol (prop) as measured by enzyme-linked immunosorbent assay (see Methods *)*. Error bars show 95% CI of triplicate measurements in a representative experiment. The mean baseline cAMP concentration in control erythrocytes was 0.61 pmol cAMP (95% CI 0.20–1.01 pmol; three experiments) per 1 × 10^8^ cells. Induced cAMP levels can vary—the maximal value obtained (once) was 14.58 pmol cAMP (95% CI 13.11–16.03 pmol) per 1 × 10^8^ cells. (G) cAMP production in ghosts measured and depicted as described in (F). Error bars show 95% CI of triplicate measurements in a representative experiment. The mean baseline cAMP concentration in control ghosts was 1.12 pmol cAMP (95% CI 0.59–1.64 pmol; three experiments) per 1 × 10^8^ cells. The maximal isoproterenol-induced value obtained in a single ghost cAMP assay was 9.00 pmol cAMP (95% CI 7.37–10.62 pmol) per 1 × 10^8^ cells.

### Estimation of Protein and ATP Concentration

Protein concentrations were measured by bicinchonic acid assay (Pierce) or by Bradford assay (Bio-Rad, http://www.bio-rad.com). The concentrations of ATP in ghosts and in normal intact erythrocytes were measured using an ATP-determination kit (A22066, Invitrogen) according to the manufacturer's instructions; for each reaction, 1–10 × 10^5^ cells were used per sample.

### Protease-Protection Assay

Ghosts (5 × 10^7^ cells per reaction) loaded with 40 μM purified GST were incubated in a total volume of 100 μl of PBS; samples were treated with or without 100 μg/ml of proteinase K (Roche, http://www.roche.com) and/or 0.5% Triton X-100 (w/v). Samples were incubated for 30 min at 37 °C, before protease activity was stopped by adding phenylmethylsulfonyl fluoride (1 mM final, Invitrogen). Proteins from 1 × 10^7^ cells were solubilized in SDS-PAGE sample buffer, before being separated by 12% acrylamide SDS-PAGE and immunoblotted with GST-specific antibodies, as previously described [4,5].

### Flow Cytometry

Ghosts loaded or unloaded with 0.5 mg/ml of 70-kDa FITC-dextran were washed three times and resuspended to 1% Hct in PBS-glucose. FITC fluorescence was measured on the gated erythroid population using a FACS Caliber flow cytometer (Becton-Dickinson, http://www.bd.com).

### Cyclic Adenosine Monophosphate Assays

Normal, intact erythrocytes or erythrocyte ghosts were incubated in cRPMI containing media alone, 10 μM isoproterenol, 10 μM propranolol-DL, or both drugs in combination, for 30 min at 37 °C in 24-well tissue-culture plates. Cells were subsequently washed twice in cold PBS-glucose. Cyclic adenosine monophosphate (cAMP) levels were measured from 2.5–5.0 × 10^8^ normal erythrocytes or ghosts using a quantitative, colorimetric cAMP immunoassay kit (Assay Designs direct cAMP immunoassay kit 900–066, http://www.assaydesigns.com). Measured cAMP concentrations were multiplied by 8.5 to account for the dilution of 50-μl cell pellets into 0.1 M HCl lysis buffer. Data are expressed as pmol cAMP per 1 × 10^8^ cells.

### Parasite Culture

P. falciparum strains 3D7 and FCB were cultured and synchronized using standard techniques [11,12]. For protein export experiments, P. falciparum 3D7 expressing P. falciparum histidine-rich protein II (PfHRPII) fused to GFP was cultured as previously described [13].

### Invasion and Growth Assays in Culture

Mature schizont-stage cultures were synchronized using standard Percoll density gradients. Uninfected ghosts were incubated in cRPMI at 2%–5% Hct with 1%–3% synchronized schizonts in 24-well plates under standard culture conditions; ghosts were loaded with fusion proteins or bathed in peptides as appropriate. When adrenergic-acting drugs were used, they were dissolved in serum-free RPMI at 100-fold final concentration. Peptides were prepared as described above. For invasion assays, Giemsa-stained thin blood smears were made at 0 and 12–18 h post-invasion to determine parasitemias. For morphological growth assays, ghosts or normal erythrocytes were combined with synchronized schizont-stage parasites and cultured as above; subsequent trophozoite- and schizont-stage growth and reinvasion was monitored by Giemsa-stained smears at 32, 44, and 64 h after invasion. To test the reinvasion of normal erythrocytes by malaria parasites in ghost cultures, parasites were cultured in either ghosts or normal erythrocytes for 60 h and then diluted into normal erythrocytes for an additional 50 h; cultures were fed with fresh cRPMI and assessed by Giemsa-stained blood smears daily.

### Microscopy

Ghost preparations were evaluated for cell morphology and loading efficiency by light and fluorescence microscopy, respectively. Parasitemias were determined by counting Giemsa-stained thin blood smears; the counter was blinded to sample identities. In experiments with ghosts, parasitemias were corroborated by counting fluorescently labeled ghosts with Hoechst 33342–stained nuclei. Light microscopy and image collection were carried out using a Zeiss Axioskop upright microscope and Nuance spectral camera/un-mixing system (Cambridge Research and Instrumentation, http://www.cri-inc.com). Fluorescence microscopy and digital image collection were carried out using an Olympus IX inverted fluorescence microscope and a Photometrix cooled CCD camera (CH350/LCCD) driven by DeltaVision software (Applied Precision, http://www.api.com) as described [6].

### Effect of β-Blockers on Growth of P. falciparum In Vitro Cultures

The inhibitory effects of adrenergic antagonists were measured using standard [^3^H]-hypoxanthine–incorporation assays [14]. The data were regressed to transition functions [15] using TableCurve 2D software (Systat, http://www.systat.com); the Lorentzian cumulative transition function was consistently the best-fit function. The effects of β-blocker–containing drug combinations on growth were tested using fixed-ratio drug-combination assays [16]. Briefly, propranolol, chloroquine, and/or artemisinin were serially diluted in 200 μl of RPMI and added to 96-well plates with 2 × 10^7^ infected erythrocytes at 0.6% parasitemia, containing primarily mature parasites. Cultures were incubated for 24 h under standard conditions until reinvasion occurred. Following invasion, 0.5 μCi of [^3^H]-hypoxanthine (Amersham Biosciences, http://www.amershambiosciences.com) was added to each well, and samples were re-incubated for 18 h. Plates were harvested onto filters, dried, and assayed for [^3^H]-hypoxanthine. Individual 50% inhibitory concentration (IC_50_) values were determined for each drug singly, and those values were used to establish drug ratios used in combination studies. Combined IC_50_ values were determined for each drug in combination at 1:0, 3:1, 1:1, 1:3, and 0:1 ratios. A fractional IC_50_ value (IC_50_ in combination/IC_50_ alone) was calculated for each drug-combination ratio and used to plot isobolograms [17]. The fractional IC_50_ values for each drug were added; a sum >1 indicates synergism, <1 indicates antagonism, and equal to 1 indicates additive effects.

### Effect of Propranolol on Sorbitol Sensitivity of P. falciparum-Infected Erythrocytes

Incubation of 0–4 h rings at 10% parasitemia was performed in 0.75 ml of 2 or 10 μM propranolol in cRPMI, or in absence of the drug, for 30 h in 24-well plates; drug and media were refreshed after 15 h. The contents of each well were washed once in PBS-glucose and resuspended in 0.75 ml of 5% sorbitol for 15 min at 37 °C (cells were gently resuspended every 5 min throughout the incubation). At 0 and 15 min, 0.25-ml samples were removed, centrifuged for 5 min at 600 *g*, and 0.15 ml of the supernatant was transferred to a well in a 96-well plate to measure hemoglobin release by spectrophotometric absorbance at 570 nm. The remainder of the sample (0.25 ml) was lysed by addition of 5 μl of 10% saponin (w/v), and the absorbance of total hemoglobin released was measured at 570 nm.

### Effect of Propranolol on Protein Export in P. falciparum-Infected Erythrocytes

Ring-stage cultures (10% parasitemia) of P. falciparum 3D7 expressing PfHRPII-GFP [13] were treated with propranolol as described above for sorbitol assays. Drug-treated cultures were grown for an additional 30 h, transferred to tubes and washed twice with PBS-glucose. Cells were imaged live by digitized fluorescence microscopy to visualize export of the tagged transgene to the infected erythrocytes [13]. Protein export to the erythrocyte was quantified from 200 optical sections using previously described methods [18].

### Effect of Propranolol/Chloroquine Drug Combination in a P. berghei Infection of Mice

Mouse experiments were performed with BALB/c mice and P. berghei ANKA strain using a standard 4-d Peter's test [19]. The IC_50_ of propranolol had been found previously to be ~8 mg/kg/d [7]; the IC_50_ of chloroquine in a chloroquine-sensitive P. berghei strain is ~1.3–1.7 mg/kg/d [19]. Mice received 1 × 10^7^ parasites intraperitoneally on day 0, followed by daily (chloroquine) and/or twice daily (propranolol) intraperitoneal drug treatments on days 0–3. Treatment groups were: control; chloroquine alone; propranolol alone; 2:1 propranolol:chloroquine; 1:1 propranolol:chloroquine; and 1:2 propranolol:chloroquine. Each group consisted of five different treatment doses with five mice per treatment dose; the control group contained five mice. Individual IC_50_ values were previously determined for each drug singly, and those values were used to establish the drug ratios used in subsequent studies. The range of chloroquine doses was 0.5–7.0 mg/kg/d; the range of propranolol doses was 1–96 mg/kg/d. Blood smears were obtained by tail bleeding on day 4, and animals were sacrificed. Combined and fractional IC_50_ values were determined for the indicated ratios, and an isobologram was plotted as for the in vitro results.

### Statistics

Results represent the mean ± standard deviation unless otherwise stated. All experiments were performed in triplicate unless stated otherwise. Where indicated, representative data from individual experiments are shown. Statistical significance was determined using the Student's *t*-test.

## Results

### Production of Ghosts with G_s_ Signaling and Malarial Infection that Closely Mimic Normal Erythrocytes

As summarized in Methods and in Figure 1, ghosts were prepared by dialysis of erythrocytes in a low MWCO membrane, simultaneously equilibrated with cargo and then resealed in a potassium-rich buffer in the presence of ATP (1 mM) and GTP (1 mM), and finally washed successively in RPMI and cRPMI and used as described below. Non-outdated blood was used to make erythrocyte ghosts; freshly drawn erythrocytes were used for cAMP and malarial infection assays. These resealed ghosts morphologically resembled normal biconcave erythrocytes (Figure 2A). Early reports suggested that “white” ghosts depleted of hemoglobin remain permeable to small molecules [9,10]. However, ghosts prepared by our procedure contained 40%–50% of starting hemoglobin and maintained the millimolar levels of ATP in which they were prepared, indicating that, at a minimum, they retained critical energy pools. These levels were half of those measured in intact erythrocytes (Figure 2B), but within the range of ATP levels reported for erythrocytes [20,21]. Clinical hematogram analysis of a ghost preparation suggested that they contained less hemoglobin (lower MCH), were slightly microcytic (lower MCV), and were more heterogeneous (higher RDW) than normal, intact erythrocytes (Figure 2B). The ghosts could be efficiently and homogenously loaded with a variety of experimental cargoes, such as high molecular weight rhodamine-labeled antibody (Figure 2C), as well as with smaller cargoes (not shown) such as low molecular weight LY (457 g/mol), 11-residue FITC-labeled G_s_ and G_scr_ peptides, 10-kDa LY-D, and 70-kDa FITC-dextran. Analyses of ghosts loaded with 70-kDa FITC-dextran by flow cytometry indicated that >99% of all cells were homogenously loaded with cargo (Figure 2D). Further tests of loading and retention of purified GST suggested that proteinaceous cargoes at 10–20 μM could be reliably encapsulated within ghosts and retained under culture conditions; this GST was resistant to degradation by exogenous protease (Figure 2E). These results indicated that ghosts stably maintain nucleotide pools and loaded cargoes.

One of our major interests in developing reconstituted erythrocyte ghosts was to study β-adrenergic signaling and its regulation by G_s_. This investigation required the presence of active β-adrenergic signaling in ghosts. As shown in Figure 2F and 2G, cAMP production stimulated in ghosts by 10 μM isoproterenol (an agonist of β-ARs) was comparable to that seen in normal erythrocytes. This stimulation was quenched by the addition of equimolar racemic propranolol (the antagonist) in both cell types. Propranolol alone did not reduce basal cAMP levels in either cell type. The basal and isoproterenol-stimulated cAMP levels were similar to those previously measured in erythrocytes [22,23]. These data show that the β-adrenergic signaling pathway is active in these ghosts.

As we were also interested in testing the effects of signaling on malarial infection in ghosts, we next characterized ghosts for their ability to support malarial infection. The intraerythrocytic lifecycle of malaria begins when extracellular merozoites rapidly (<2 min) invade mature erythrocytes. Intracellular ring-stage parasites result and subsequently enlarge into trophozoites. Schizogony follows when an average of 16 daughter parasites assemble and finally lyse out of infected cells to invade new erythrocytes. Therefore, ring formation provides a measurement of invasion and early development, while the appearance of trophozoites and schizonts indicates parasite maturation within the erythrocyte. In *P. falciparum,* this intracellular cycle lasts 48 h.

**Figure 3:**
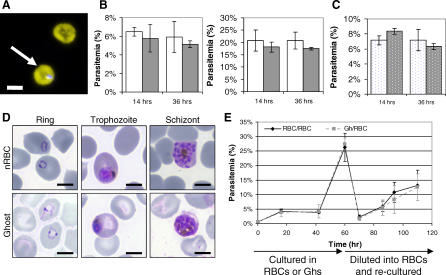
Erythrocyte Ghosts Support Malarial Invasion and Growth (A) LY-D–loaded erythrocyte ghosts (yellow) were infected with P. falciparum (detected by blue DNA stain Hoechst 33342; indicated by arrow). Bar represents 5 μm. (B) Ghosts (white bars) or erythrocytes (grey bars) infected at low (left) or high (right) parasitemias were monitored by Giemsa staining of thin blood smears at the indicated times of infection; mean values are shown. Error bars show standard deviation of triplicate measurements of a representative experiment. (C) An infected culture containing 50% ghosts and 50% erythrocytes was monitored for cell type-specific parasitemias by counting the number of Hoechst 33342–stained parasite nuclei in fluorescent ghosts (white bars) versus non-fluorescent erythrocytes (grey bars) using DIC and fluorescence microscopy of live cells (see Methods). Error bars show standard deviation of triplicate measurements of a representative experiment. (D) Giemsa-stained thin blood smears showing ring-, trophozoite-, and schizont-stage parasites in normal (upper) and ghosted (lower) erythrocytes. Bar represents 5 μm. (E) Growth of P. falciparum in ghosts (Ghs) or in erythrocytes (RBCs) in culture over multiple life cycles. Parasites were cultured in ghosts (indicated by dashed line) or in normal erythrocytes (indicated by solid line) for 60 h to ~25% parasitemia, and then were diluted into normal erythrocytes and grown for an additional 50 h. Parasitemia was assessed by counting Giemsa-stained thin blood smears. Error bars show 95% CIs of duplicate measurements at each time point.

As shown, the resealed ghosts were infected by P. falciparum (Figure 3A). Remarkably, infection occurred with the same efficiency as in normal erythrocytes, and ghosts supported normal parasite maturation at both low and high parasitemias (Figure 3B). In cultures containing equal numbers of ghosts and normal erythrocytes, no preference was observed for invasion or growth in either cell type (Figure 3C). Furthermore, parasites in ghost cultures appeared morphologically similar to their counterparts in normal erythrocytes at the three distinct ring, trophozoite, and schizont stages (Figure 3D). Schizonts that mature in ghosts are capable of rupturing and reinvading ghosts (Figure 3E). Furthermore, parasites grown in either ghosts or normal erythrocytes when sub-cultured into normal erythrocytes showed no difference in growth over the next infective cycle (Figure 3E). Over three life cycles, completion of maturation of parasites in ghosts can extend from 48 to 54 h per cycle (unpublished data). We do not know why this occurs, but it is within an acceptable range of the asexual life cycle in normal P. falciparum cultures.

Collectively, these data show that erythrocyte ghosts, active in supporting β-adrenergic signaling and malarial invasion and growth, can be produced and stably loaded with exogenous cargo. Remarkably, the ghosts are essentially equivalent to normal erythrocytes in a number of critical respects, suggesting they may be useful for investigating multiple functions of the human erythrocyte and its infection by malaria parasites.

### Targeting Erythrocyte G_s_

**Figure 4:**
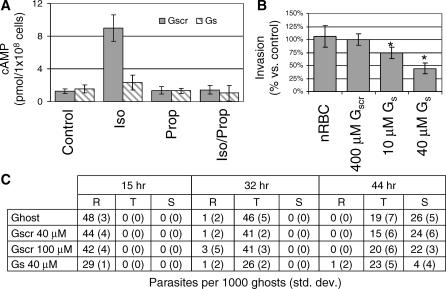
Targeting Erythrocyte G_s_ Down-Regulates β-Adrenergic Signaling and Inhibits Malarial Infection (A) Ghosts incubated in 40 μM G_s_ (indicated by hatched bars) or G_scr_ control peptide (indicated by solid bars) were treated with agonist (isoproterenol [iso]) and/or antagonist (propranolol [prop]), and cAMP was measured as described in Methods. Error bars show 95% CI of triplicate measurements from a representative experiment of three independent experiments. The mean baseline cAMP concentration in control ghosts was 1.08 pmol cAMP (95% CI 0.57–1.60 pmol; four experiments) per 1 × 10^8^ cells. The maximum isoproterenol-induced cAMP level obtained in control-treated ghosts was 12.45 pmol cAMP (95% CI 10.20–14.70 pmol) per 1 × 10^8^ cells. (B) Ghosts incubated with G_s_ peptide (≤40 μM) or G_scr_ control peptide (up to 400 μM) were assayed for malarial invasion. Data are represented as percentage inhibition relative to control cultures treated with equimolar concentrations of control peptide. Three experiments in triplicate for each data point except 10 μM (two experiments in triplicate). Error bars show 95% CI for all experiments; asterisks indicate *p* < 0.001 compared to 400 μM control G_scr_ peptide–treated cultures. (C) Parasites in ghosts were incubated with the indicated concentrations of peptide and monitored at 15, 32, and 44 h after invasion by examination of Giemsa-stained thin blood smears. The number of ring (R)–, trophozoite (T)–, and schizont (S)–stage parasites per 1,000 total ghosts at each time point are shown; numbers in parentheses indicate the standard deviation of triplicate measurements from one experiment; a total of two experiments were carried out.

Our previous studies had shown that a peptide designed to inhibit G_s_ protein interaction with its receptors blocked malarial entry into erythrocytes [7]. However, since the peptide does not diffuse into normal erythrocytes, there was no evidence that it blocked endogenous G_s_ function in the red cell. In addition, erythrocytes are terminally differentiated cells, their signaling mechanisms are not well understood, and direct evidence that erythrocyte G proteins are active is not available. We therefore introduced the G_s_ peptides into ghosts to establish whether it acted on its designated target G_s_. As shown in Figure 4A, G_s_ peptide blocked cAMP production in response to the β-AR agonist, isoproterenol. The extent of this inhibition was comparable to that seen with propranolol, a β-adrenergic antagonist that blocks G_s_ signaling. This inhibitory effect was achieved at 40 μM G_s_ peptide, which is known to be effective at 10–50 μM in other cell types [24]. In contrast, the control scrambled peptide (G_scr_) had no effect on cAMP production stimulated by isoproterenol. These data establish that β-adrenergic signaling in erythrocytes is mediated through G_s_ and that the G_s_ peptide directly targets and blocks activation of erythrocyte G_s_.

To directly link this inhibition of host G_s_ to malarial infection, we assayed the effect of the peptide on parasite invasion and intracellular maturation in ghosts. As shown in Figure 4B, G_s_ peptide blocked malarial invasion at low micromolar concentrations. Importantly, there was a marked reduction in infection at the same peptide concentration (40 μM) that blocked G_s_ activation. In contrast, control G_scr_ peptide had no effect on parasite invasion into ghosts, even at a higher concentration (up to 400 μM, Figure 4B). In addition, peptides designed to block G_i_ or G_q_ proteins also had no effect on invasion (Figure S1). These data establish that the G_s_ peptide can block malarial entry at the same concentrations that quench receptor-mediated G_s_ signaling. However, invasion is not completely abolished, suggesting that there may be additional mechanisms that regulate entry. Thus, if G_s_ only regulates invasion, it may not provide a satisfactory target for development of antimalarials.

Our results presented in Figure 3 suggest that ghosts support normal invasion and intracellular development of parasites, and thus allow us to examine erythrocyte functions needed in malarial entry as well as intracellular growth. To determine the requirement of G_s_ signaling for intraerythrocytic parasite growth, we followed the development of rings formed in the presence of 40 μM peptide to schizogony. As shown in Figure 4C, at this concentration of peptide, there was no measurable effect on trophozoites formed, but the number of schizonts detected was reduced. Since maturing trophozoites are known to actively ingest as much as 80% of the erythrocyte cytoplasm, the net intraerythrocytic peptide concentration at schizont stages of growth may be lower. Nonetheless, the data presented in Figure 4B and 4C provide a pharmacological link between the inhibition of host erythrocyte signaling, malaria parasite invasion, and intraerythrocytic development.

**Figure 5:**
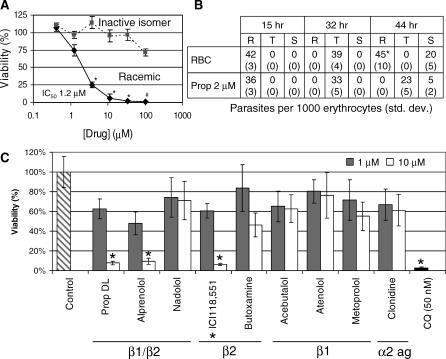
β-Blockers Inhibit Maturation of *P. falciparum* in In Vitro Cultures **(**A) [^3^H]-hypoxanthine incorporation of P. falciparum 3D7 when treated with racemic propranolol (indicated by black diamonds) or its inactive isomer (indicated by grey squares). Error bars show the standard deviation of triplicate measurements. IC_50_ values were determined by fitting the data as described in Methods; the IC_50_ value obtained for racemic propranolol (1.2 μM; 95% CI 1.0–1.6 μM) is shown. Three experiments; hatched sign indicates *p* < 0.001; asterisks indicate *p* < 0.03 compared to equimolar inactive propranolol. (B) Effect of 2 μM propranolol on intracellular growth in normal erythrocytes. Mock- and drug-treated cultures were monitored at 15, 32, and 44 h after invasion by examination of Giemsa-stained thin blood smears. The number of ring (R)–, trophozoite (T)–, and schizont (S)–stage parasites per 1,000 total erythrocytes at each time point are shown; numbers in parentheses indicate the standard deviation of triplicate measurements from one experiment; the total number of experiments was three. (C) [^3^H]-hypoxanthine incorporation of P. falciparum 3D7–infected erythrocytes treated with 1 or 10 μM concentrations of adrenergic-acting drugs (nonspecific β_1_/β_2_–antagonists: propranolol, alprenolol, and nadolol; β_2_-specific antagonists: ICI118,551 and butoxamine; β_1_-specific antagonists: acebutalol, atenolol, and metoprolol; and α_2_-specific agonist [ag]: clonidine). Chloroquine (50 nM) was used as an inhibitory control. Triplicate samples; asterisks denote a treatment with *p* < 0.001 compared to control-treated cultures.

A second, independent way of down-regulating G_s_ signaling in erythrocytes is by using β-antagonists. The β-blocker propranolol was previously known to inhibit isoproterenol-mediated stimulation of invasion at ~10 μM [7], but effects on P. falciparum intracellular maturation and growth were not studied. In standard growth assays, we found that propranolol inhibited [^3^H]-labeled hypoxanthine uptake by half (IC_50_) at 1.2 μM and by 90% inhibitory concentration (IC_90_) at 7.1 μM (Figure 5A). Since hypoxanthine incorporation is a standard measure of parasite proliferation, the data confirm that, in addition to invasion, propranolol can block one or more processes needed for intracellular parasite survival. The inactive isomer of propranolol was not inhibitory, suggesting that the antimalarial effect of racemic propranolol is due to specific down-regulation of erythrocyte G_s_ signaling. Examination of Giemsa-stained slides from treated cultures (Figure 5B) showed that 2 μM propranolol blocked intracellular maturation of parasites to the schizont stage. At 2 μM, propranolol had no significant effect on invasion, which is consistent with our earlier data indicating that significant inhibition of invasion required ~10 μM drug [7]. Hence, the observed IC_50_ of 1.2 μM suggests that most of the inhibitory activity of propranolol is due to blockage of intracellular parasite maturation. Other β_2_-antagonists such as ICI118,551 and alprenolol also blocked parasite growth, as measured by hypoxanthine incorporation (Figure 5C). One β_1_/β_2_–antagonist, nadolol, was ineffective at 10 μM. All three β_1_-specific antagonists had no effect, and this may reflect a lack of β_1_-adrenergic receptors on the erythrocyte. The three most active β-antagonists (including propranolol) appear to show IC_50_ values of between 1 and 10 μM. Testing additional β-antagonists (particularly β_2_ antagonists) may yield additional active inhibitors.

**Figure 6:**
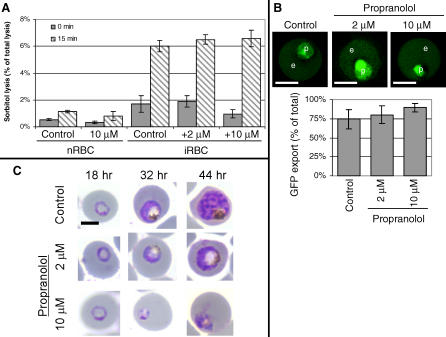
Analysis of the Maturation Defect in Propranolol-Treated Parasites (A) Effect of propranolol on sorbitol sensitivity of infected erythrocytes. Normal or infected erythrocytes were mock-treated or incubated with 2 or 10 μM propranolol for 30 h and then resuspended in 5% sorbitol as described in Methods. Bars represent percentage lysis as calculated by the hemoglobin absorbance (570 nm) of the supernatant after 0 and 15 min of sorbitol treatment divided by the hemoglobin absorbance of a saponin-treated sample (where 100% of hemoglobin is released) from the same culture. Error bars show the standard deviation from triplicate samples. (B) Effect of propranolol on export of a parasite-encoded protein (PfHRPII) fused to GFP to the host erythrocyte. Fluorescence micrographs of infected erythrocytes that were mock-treated or exposed to 2 or 10 μM propranolol for 30 h (see Methods) are shown, with single optical sections. Bar represents 5 μm. The fraction of GFP exported to the erythrocyte (e) from the parasite (p) was determined (see Methods) and is relatively unchanged by drug treatment. Error bars show the standard deviation from ten samples per treatment. (C) Giemsa-stained thin blood smears showing the morphology of parasites when infected erythrocytes were treated with 2 or 10 μM propranolol for 15, 32, and 44 h of culture. Infected erythrocytes treated with 2 μM propranolol showed significant retardation in schizont formation by 44 h. At 10 μM propranolol, a growth defect was observed by 32 h post-invasion. Bar represents 3 μm; all images are displayed at the same scale.

To examine intraerythrocytic process(es) that may be inhibited by propranolol treatment, we tested several general functions of malaria parasites. First, we tested the sensitivity of control and propranolol-treated parasites to lysis in the presence of 5% sorbitol, which is known to be actively imported into trophozoite- and schizont-stage parasites—likely via the plasmodial nutrient transport channel PESAC [25]. No difference was found between control and drug-treated parasites (Figure 6A), suggesting that maturation to the trophozoite stage occurred in both 2 and 10 μM propranolol-treated infected cells. Second, export of a parasite-encoded protein PfHRPII fused to GFP to the erythrocyte [13] was not blocked by either 2 or 10 μM propranolol (Figure 6B), suggesting that the drug did not act by altering protein export to host cells. Nonetheless, examination of the morphology of 2 μM propranolol-treated parasites at 44 h post-invasion (Figure 6C) revealed a lack of the segmented, mature schizonts characteristically found in control cultures at 44 h. Control cultures also showed the presence of new rings (marked by an asterisk in Figure 5B) indicating their acceleration past segmented schizonts to new rings. (This variation of the 48-h intraerythrocytic cycle is frequently observed in culture.) The effect of propranolol on parasite morphology was greater at 10 μM (compared to 2 μM), but the precise nature of one or more steps regulated by G_s_ signaling and needed for maturation to the schizont stage remains unknown.

### Inhibitors of Erythrocyte G_s_ Signaling May Be Useful as Novel Antimalarial Therapeutics

**Figure 7:**
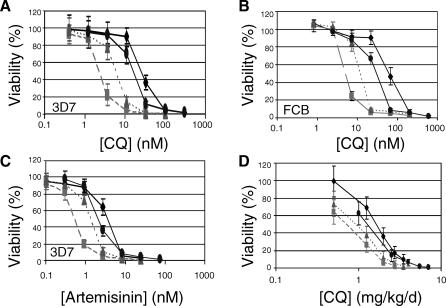
Propranolol Inhibits Malarial Growth in Combination with Existing Antimalarial Drugs (A) [^3^H]-hypoxanthine incorporation (see Methods) of P. falciparum 3D7 treated with chloroquine alone (CQ, indicated by black diamonds) or with ratios of both drugs (CQ:propranolol, 3:1 [black spheres]; 1:1 [grey triangles]; and 1:3 [grey squares]). Error bars show standard deviation of triplicate samples. IC_50_ values were determined by fitting the data as described in Methods. IC_50_ CQ alone: 28.9 nM (95% CI 22.3–36.6 nM). The IC_50_ CQ values when treated with combinations of both drugs in different ratios of CQ:propranolol were as follows: 3:1, 14.7 nM (95% CI 11.9–18.1 nM); 1:1, 5.9 nM (95% CI 4.9–7.0.6 nM); and 1:3, 2.9 nM (95% CI 2.5–3.2 nM). (B) [^3^H]-hypoxanthine incorporation of P. falciparum chloroquine-resistant FCB strain treated with chloroquine alone (CQ, indicated by black diamonds) or with ratios of both drugs (CQ:propranolol, 3:1 [black spheres]; 1:1 [grey triangles]; and 1:3 [grey squares]). Error bars show standard deviation of triplicate samples. IC_50_ values were determined by fitting the data as described in Methods. IC_50_ CQ alone: 63.2 nM (95% CI 52.4–76.4 nM). The IC_50_ CQ values when treated with combinations of both drugs in different ratios of CQ:propranolol were as follows: 3:1, 26.7 nM (95% CI 24.6–29.0 nM); 1:1, 9.7 nM (95% CI 9.3–10.1 nM); and 1:3, 6.0 nM (95% CI 5.8–6.2 nM). (C) [^3^H]-hypoxanthine incorporation of P. falciparum 3D7 treated with artemisinin alone (black diamonds) or with ratios of both drugs (artemisinin:propranolol, 3:1 [black spheres]; 1:1 [grey triangles]; and 1:3 [grey squares]). Error bars show standard deviation of triplicate samples. IC_50_ values were determined by fitting the data as described in Methods. IC_50_ artemisinin alone: 3.2 nM (95% CI 2.9–3.6 nM). The IC_50_ artemisinin values when treated with combinations of both drugs in different ratios of artemisinin:propranolol were as follows: 3:1, 2.3 nM (95% CI 2.0–2.6 nM); 1:1, 1.3 nM (95% CI 1.2–1.4 nM); and 1:3, 0.56 nM (95% CI 0.5–0.6 nM). (D) Combinations of propranolol with chloroquine (CQ) in a 4-d Peter's test of P. berghei ANKA–infected Balb/c mice (see Methods). IC_50_ CQ alone: 1.6 mg/kg/d (95% CI 1.2–2.2 mg/kg/d). The IC_50_ CQ values when treated with combinations of both drugs in different ratios of CQ:propranolol were as follows: 2:1, 1.2 mg/kg/d (95% CI 1.0–1.4 mg/kg/d); 1:1, 1.0 mg/kg/d (95% CI 0.8–1.3 mg/kg/d); and 1:2, 0.7 mg/kg/d (95% CI 0.4–1.0 mg/kg/d). Error bars show standard deviation of measurements from five mice per treatment group.

The emergence of drug-resistant parasites has led to the need for combinations of two or more antimalarials to improve microbial killing and reduce further resistance. Propranolol was therefore combined and tested with two widely used antimalarials, chloroquine and artemisinin. IC_50_ values were first independently determined for each individual drug in P. falciparum 3D7 and/or FCB strains (Figure S2). Using those values, fixed ratios of propranolol:chloroquine (or propranolol:artemisinin) were tested by in vitro hypoxanthine assays [16]. When used in combination against P. falciparum strain 3D7, propranolol reduced the amount of chloroquine required to achieve an IC_90_ dose by >1 log (Figure 7A and Table S1; the 3:1 and 1:1 propranolol:chloroquine combinations had 11- and 4-fold effects, respectively). Isobologram analysis (Figure S3) indicated that there was an additive antimalarial effect between propranolol and chloroquine. Similar results were found for this combination against the more resistant P. falciparum FCB (Figures 7B and S3). Propranolol did not reverse chloroquine resistance, but did reduce the effective dose in the resistant (FCB) and sensitive (3D7) strains. This result was different than the well-known chloroquine reversal seen in previous fixed-ratio drug combinations using chloroquine and the calcium-channel blocker verapamil [16]. Importantly, this β-blocker did not antagonize chloroquine against either strain, suggesting that blocking G_s_ function may enable utilization of chloroquine at lower concentrations and thus may facilitate its use against resistant parasites.

Artemisinin is a newer, schizonticidal antimalarial that is gaining acceptance for treatment of severe and uncomplicated malaria because of its rapid action and lower prevalence of resistance [26,27]. Its mode of action is thought to be distinct from that of chloroquine [28]. When used in combination, propranolol also reduced the amount of artemisinin required to achieve an effective dose 5-fold (Figure 7C) and acted in a potent, additive fashion (Figure S3; Table S1). Since propranolol was additive with two distinct drugs, it may be effective when combined with a range of existing antimalarials in order to also reduce their required doses.

Our previous results suggested that the requirement for host signaling via β-ARs and G_s_ was conserved across parasite species. We therefore further tested propranolol-containing drug combinations in the P. berghei ANKA mouse model of malarial infection. When tested alone in mice, the IC_50_ of racemic propranolol was 7.5 mg/kg/d [7]. As shown in Figures 7D and S3, a combination of 2:1 propranolol:chloroquine reduced the IC_50_ of chloroquine from 1.64 to 0.66 mg/kg/d. The 1:1 and 1:2 propranolol:chloroquine combinations had less effect. This finding is in contrast to our results in vitro where 1:1 propranolol:chloroquine combinations had a 4-fold effect. This discrepancy may be due to the fact that the half-life of propranolol is 3–4 h [29,30], and therefore multiple treatments and/or more stable compounds may prove more effective. Furthermore, since mice metabolize drugs at higher levels than humans [31,32], mouse-derived data likely overestimate the amount of drug required in humans. Importantly, as in in vitro studies, the combinations tested were not antagonistic (Figure S3)*.* Thus, both our in vitro and in vivo data suggest that targeting erythrocyte G_s_ in combination with existing antimalarial drugs may have therapeutic potential and may reduce the amount of existing drug needed to effectively treat patients.

### Discussion

In this paper, we describe the development of erythrocyte “ghosts” active in β-adrenergic signaling that support robust P. falciparum growth in culture. With this system, we show that a G_s_ peptide inhibits activation of erythrocyte β-AR signaling by β-agonists, providing definitive evidence that the peptide targets erythrocyte G_s_ signaling. At the same pharmacological concentrations, the peptide significantly inhibits malarial invasion of ghosts as well as intracellular maturation of parasites. Furthermore, we find that inhibiting G_s_ signaling with β-blockers also inhibits intracellular parasite growth. Finally, we show that a β-blocker such as propranolol can be used to reduce doses of existing antimalarials in both in vitro and in vivo infections. We therefore establish that G_s_ offers a host target to fight infection and to develop new antimalarial chemotherapies.

Erythrocyte ghost preparations have been described previously for studying the erythrocyte membrane and cytoskeleton and malarial infection [33–38]. However, malarial infection rates of earlier ghosts were either untested or lower than in normal erythrocytes. In contrast, the ghosts described here retained mature erythrocyte double-discoidal morphology, G_s_ signaling responses, and support of robust malarial infection. Several differences between our procedure and procedures described in prior reports include the lysis of erythrocytes in 3.5-kDa MWCO dialysis membranes rather than in 12–14-kDa MWCO membranes used previously, and our use of a potassium-rich resealing buffer rather than isotonic saline. Dialysis pore size may be critical since a 10–13-kDa dialyzable, cytoplasmic erythrocyte protein was reported necessary for malarial invasion of ghosts [39,40]. Prior studies did not include GTP, which was incorporated here to ensure the support of a GTP cycle. Ghosts prepared in this manner can be loaded with a variety of peptidic, proteinaceous, fluorescent, or other cargoes and are likely suitable for studying many aspects of erythrocyte function and malarial infection. We have used them to definitively establish that erythrocyte β-adrenergic signaling is indeed mediated by G_s_ and thus to explain why inhibitors of receptor-mediated G_s_ signaling (such as propranolol) block malaria parasite invasion and intracellular growth. The findings strongly support the possibility that the erythrocyte G_s_ pathway might be targeted to treat malaria.

The concept of host-targeted therapies for treating infectious diseases has only emerged recently and, to the best of our knowledge, is unexplored for parasitic infections. Since malaria parasites must invade host cells to proliferate, host pathways necessary for parasite survival present opportunities for intervention. Here we have shown that targeting erythrocyte G_s_ inhibits parasite growth within erythrocytes as well as at the initial entry event. One obvious possible disadvantage of host-targeted therapy is potential toxicity to the host since G_s_ regulates many cellular pathways by modulating transcription factors, ion channels, metabolic enzymes, and other molecules [41]. However, G_s_ signaling alone is insufficient to drive vacuole formation (e.g., vacuoles do not form in isoproterenol-treated erythrocytes), and therefore parasite components are expected to engage this host signaling pathway during invasion [42]. Other parasite proteins may interact with this pathway during intracellular growth. Thus, optimal inhibitors should target interactions between host G_s_ and parasite proteins, and these may have significantly reduced toxicity, particularly if they can be made additionally selective for erythrocyte G_s_. The wide array of pharmacological GPCR-inhibitors currently available suggest chemical backbones that may be useful in rational design of such antimalarials.

In addition, antihypertensive drugs that down-regulate G_s_ via host receptors (i.e., β-blockers such as the first-generation drug propranolol) are extremely well-tolerated, partly because the host can adapt to drug treatment by producing additional adrenergic receptors and G_s_. Since the mature erythrocyte is incapable of de novo protein synthesis, it cannot mount such an adaptive response. Thus, erythrocytes may be more vulnerable to inhibition of β-AR/G_s_ signaling, while other host tissues could adapt to avoid toxicity. A well-developed, host-targeted antimicrobial therapy directed at human chemokine receptor 5 (CCR5) for the treatment of drug-resistant HIV is currently in clinical trials. Short-term treatment with CCR5 antagonists reduced viral loads in infected patients [43], providing proof of concept for targeting a host determinant to treat a major infectious disease.

Malaria is a major threat to global health, and there is an imperative to use existing drugs to treat patients while the search for vaccines and better drugs continues. Development of a new drug is expensive and can take more than a decade [44]. Propranolol and a wide range of β-blockers are currently approved for human use. In humans, racemic propranolol is typically prescribed at ~60–320 mg/d (~0.8–4.5 mg/kg/d in an average 70-kg person) [45] with a maximum clinical dose of ~10 mg/kg/d. The maximum concentration of propranolol in blood approaches 1 μM [29], and thus it is likely that treatments can be designed that achieve IC_50_s in the bloodstream (0.4–1.2 μM in the work discussed in this paper; 0.4 μM, Drug Screening Program, Strategic and Discovery Research UNICEF/UNDP/World Bank/WHO Special Programme for Research and Training in Tropical Diseases, personal communication) using a range of dosages comparable to those used for hypertension. Furthermore, higher concentrations of the drug can also be administered [45].

Malaria patients may be hypotensive [46], and therefore treatment with β-blockers must be considered carefully. However, at normal dosages, propranolol does not usually cause orthostatic hypotension because the α-adrenergic system maintains peripheral vasoconstriction [47,48]; some patients may show mild orthostatic hypotension [49]. Other quinoline antimalarials (e.g., quinine and mefloquine) may also exacerbate orthostatic hypotension in malaria patients [46,50]. The main cause of hypotension in malaria patients is acidosis secondary to hypovolemia, which can be managed with intravenous saline. Propranolol has been found to mitigate some hyper-adrenergic effects that occur in patients with shock secondary to hypovolemia. In such cases, β-adrenergic blockade by propranolol is thought to reduce muscle lactate production and to limit metabolic acidosis [51,52]. Thus, this drug may have unseen benefits for clinically ill patients.

As with any new drug, care would obviously need to be taken in any clinical malaria study using propranolol-containing combinations. Propranolol is contraindicated for use with the quinoline antimalarial quinidine [47], which also has α-adrenergic–blocking activities [53]. Other quinoline antimalarials (e.g., quinine and mefloquine) that exacerbate orthostatic hypotension also produce electrocardiographic abnormalities (i.e., long corrected QT interval), although not of the severity seen with halofantrine [54]. There is one report of a patient with a previous myocardial infarction who was taking propranolol and suffered a cardiopulmonary arrest 5 h after taking a single dose of mefloquine; the patient made a full recovery [55]. Mefloquine and propranolol are also associated independently with depression. Thus, some quinolines may be contraindicated for use with propranolol. However, only minor, asymptomatic corrected QT interval disturbances are found with other antimalarial drugs such as chloroquine (a quinoline) [56], sulfadoxine-pyrimethamine [57], and artemisinin [58]; no electrocardiographic changes were associated with atovaquone-proguanil alone or in combination with artemisinin [59]. Overall, propranolol has few serious side effects, is recommended for use in pregnant women, is widely approved by regulatory agencies, and is frequently taken for life.

In addition to its safety and efficacy, propranolol is made even more attractive by its low off-patent cost, high stability, and ease of production. In addition, second- and third-generation β-blockers have been developed, and all generations of β-blockers are routinely taken for many years to treat chronic conditions. Thus, it may be an appropriate time to evaluate combinations containing β-blockers to treat human clinical infections that fail to respond to optimized antimalarial therapy.

## Supporting Information

Figure S1 G_i_- and G_q_-Specific Peptides Do Not Inhibit Malarial Invasion of Ghosts Erythrocyte ghosts were incubated with 50 μM control G_scr_, G_s_, G_i_, or G_q_ peptides and tested for invasion as described in Methods. Error bars show standard deviations across three experiments in triplicate.
(52 KB PPT)

Figure S2 [^3^H]-Hypoxanthine Assays to Determine In Vitro IC_50_ Values of Individual Drugs Used in This Study IC_50_s for each drug were determined in chloroquine-sensitive (3D7) and chloroquine-resistant (FCB) P. falciparum by in vitro [^3^H]-hypoxanthine incorporation (see Methods). IC_50_ values were determined by fitting the data as described in Methods; each data point is shown in triplicate. Asterisks denote *p* < 0.05; hash marks denote *p* < 0.01; crosses denote *p* < 0.001. CQ, chloroquine; Art, artemisinin; Prop, propranolol.
(127 KB PPT)

Figure S3 Propranolol Functions Additively with Existing Antimalarials In Vitro and In Vivo Isobolograms (see Methods) are presented for in vitro studies of propranolol and chloroquine ([A], in 3D7 strain; [B], in FCB strain) and propranolol and artemisinin ([C], in 3D7 strain), and an in vivo study of propranolol and chloroquine in the P. berghei ANKA mouse model of malarial infection (D).
(184 KB PPT)

Table S1 Effects of Propranolol-Containing Drug Combinations on Growth of P. falciparum and P. berghei
(20 KB XLS)

### Accession Numbers

The Swiss-Prot (http://www.ebi.ac.uk/swissprot) accession numbers for the proteins discussed in this paper are human β_2_-AR (P07550); human heterotrimeric G_i_ (P63096); human heterotrimeric G_q_ (P29992); human heterotrimeric G_s_ (P63092); and PfHRPII (P90582).

## Abbreviations

ATP: adenosine 5′-triphosphate
β-AR: β-adrenergic receptor
β_2_-AR: β_2_-adrenergic receptor
cAMP: cyclic adenosine monophosphate
CCR5: chemokine receptor 5
CI: confidence interval
cRPMI: complete RPMI-1640
DTT: dithiothreitol
FITC: fluorescein isothiocyanate
GFP: green fluorescent protein
GPCR: G-protein–coupled receptor
GST: glutathione S-transferase
GTP: guanosine 5′-triphosphate
Hct: hematocrit
IC50: 50% inhibitory concentration
IC90: 90% inhibitory concentration
LY: lucifer yellow
LY-D: lucifer yellow-dextran
MCH: mean corpuscular hemoglobin
MCV: mean corpuscular volume
MWCO: molecular weight cut-off
PfHRPII: *P. falciparum* histidine-rich protein II
RDW: red cell distribution width
RPMI: RPMI-1640
